# Laparoscopic Management of Sigmoid Colon Perforation With Transanal Extrusion of a Ventriculoperitoneal Shunt Nine Weeks After Placement: A Case Report

**DOI:** 10.7759/cureus.114080

**Published:** 2026-08-06

**Authors:** Urias Hernandez Lopez, Pastor Thomas Olivares, Elloth Tamara Contreras, Rossany Meriño Rodríguez, Cristian Barros Rada

**Affiliations:** 1 Department of Surgery, University of Sinu, Cartagena, COL; 2 Department of Medicine, University of Sinu, Cartagena, COL; 3 Department of Medicine, Rafael Nuñez University Corporation, Cartagena, COL; 4 Department of Medicine, University of Magdalena, Santa Marta, COL

**Keywords:** hydrocephalus, infant, intestinal perforation, laparoscopy, postoperative complications, ventriculoperitoneal shunt complications

## Abstract

Bowel perforation with transanal extrusion of a ventriculoperitoneal shunt is a rare and potentially serious complication. We report a 22-month-old boy with spina bifida and hydrocephalus who presented with the distal catheter protruding through the anus nine weeks after shunt placement. Laparoscopy identified a single approximately 10-mm sigmoid colon perforation without abscess, feculent contamination, or macroscopic peritonitis. Under direct visualization, the previous catheter was mobilized into the abdominal cavity and removed transanally through the existing perforation. The defect was closed primarily, followed by peritoneal lavage. Given the absence of gross contamination, neurological manifestations, or initial evidence of cerebrospinal fluid infection, a completely new shunt was placed through a separate tract during the same operation. Cerebrospinal fluid and blood cultures remained negative. The patient resumed enteral feeding on postoperative day 3, was discharged on postoperative day 5, and remained asymptomatic at the three-month follow-up. This case highlights the diagnostic and therapeutic utility of laparoscopy in selected patients.

## Introduction

Ventriculoperitoneal shunting is widely used for cerebrospinal fluid diversion in patients with hydrocephalus. Bowel perforation caused by the distal catheter is one of its rarest abdominal complications, with a reported incidence of approximately 0.1%-0.7% of shunt procedures. Although bowel perforation may occur at any age, anal migration of the distal catheter is reported predominantly in pediatric patients, possibly because of greater bowel-wall fragility and intestinal motility [1,2]. Despite its low frequency, this complication is potentially serious because the catheter may establish a communication between the intestinal lumen and the cerebrospinal fluid diversion system.

The underlying mechanism has not been fully established. Proposed factors include chronic pressure from the catheter tip, a local inflammatory and fibrotic reaction that anchors the catheter to the bowel wall, and progressive pressure necrosis leading to transmural erosion. After the catheter enters the intestinal lumen, peristalsis may propel it distally, eventually resulting in transanal extrusion [1,2].

Because of its rarity, no standardized surgical approach has been established. Management must be individualized according to the patient’s clinical condition, the presence of intra-abdominal contamination or central nervous system infection, the site of perforation, and the intraoperative findings.

We report the case of a 22-month-old child with sigmoid colon perforation and transanal extrusion of a ventriculoperitoneal shunt catheter detected nine weeks after shunt placement. A laparoscopic approach enabled identification of the perforation site, safe transanal extraction of the catheter under direct visualization, and primary suture repair of the colonic defect.

## Case presentation

**Figure 1:**
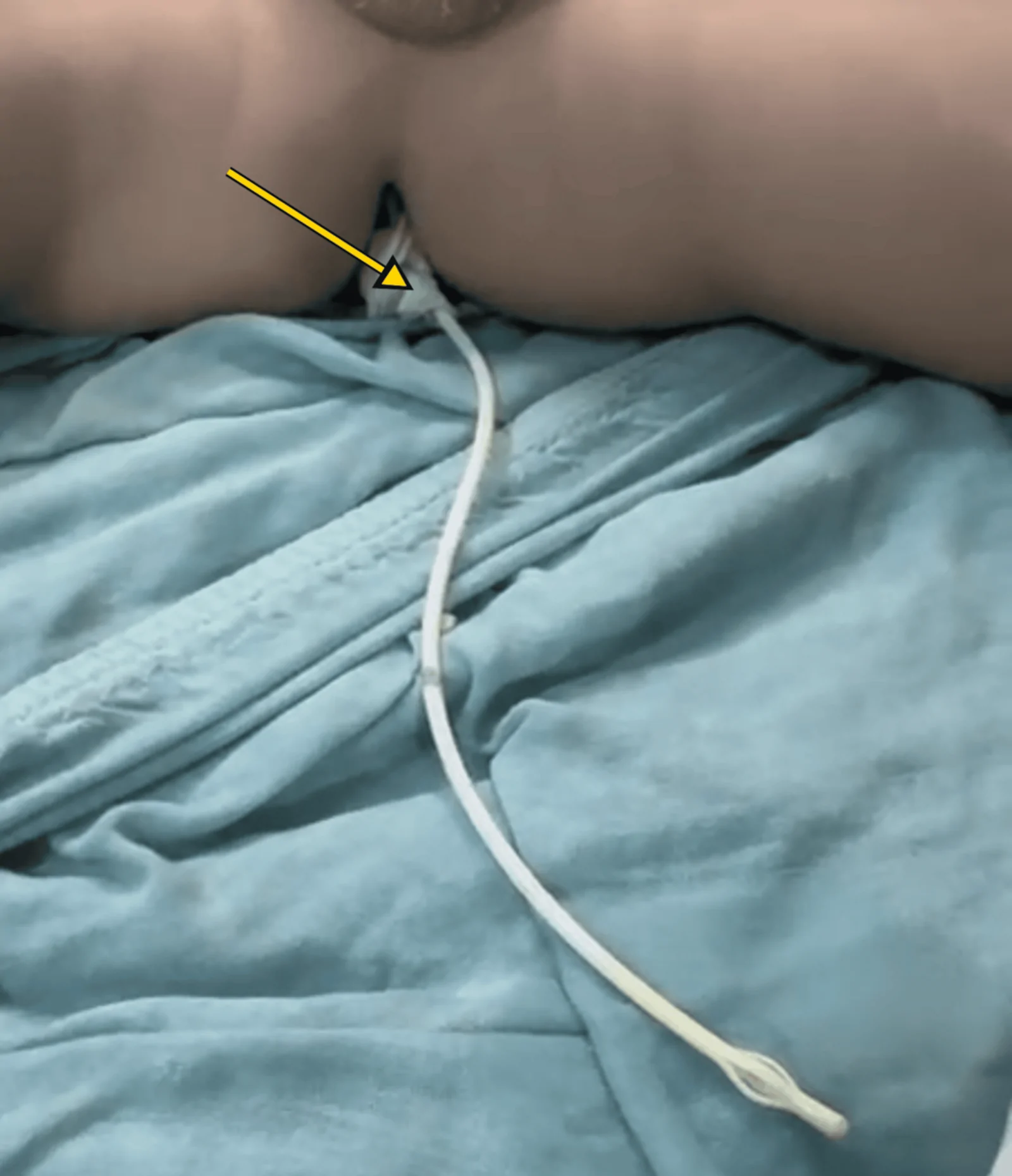
Transanal extrusion of a ventriculoperitoneal shunt catheter Clinical photograph showing the distal ventriculoperitoneal shunt catheter protruding through the anus nine weeks after shunt placement. The arrow indicates the site of transanal extrusion

A 22-month-old male child with spina bifida and hydrocephalus underwent ventriculoperitoneal shunt placement. His postoperative course was uneventful, without abdominal or neurological symptoms, and he was discharged in good clinical condition. Nine weeks later, he presented after the distal shunt catheter was observed protruding through the anus following defecation (Figure 1).

On admission, his blood pressure was 91/64 mmHg, heart rate was 115 beats/minute, respiratory rate was 29 breaths/minute, and oxygen saturation was 98% while breathing room air. Physical examination confirmed transanal extrusion of the distal catheter and revealed clinical signs of peritoneal irritation. No neurological manifestations were identified.

Laboratory testing showed a leukocyte count of 14,570 cells/mm³ with 89% neutrophils, a platelet count of 389,000 cells/mm³, and a C-reactive protein level of 36 mg/dL. Blood and cerebrospinal fluid samples were obtained before antimicrobial therapy. Cerebrospinal fluid Gram staining was negative, and both cerebrospinal fluid and blood cultures subsequently showed no microbial growth. Because transanal extrusion of the catheter was clinically evident and signs of peritoneal irritation warranted urgent surgical exploration, no additional preoperative imaging was obtained.

Laparoscopy was performed using three trocars placed at the umbilicus, left upper quadrant, and hypogastrium. A single perforation measuring approximately 10 mm was identified in the sigmoid colon, through which the distal shunt catheter had entered the intestinal lumen. No additional intestinal perforations, intra-abdominal abscesses, feculent contamination, or macroscopic peritonitis were identified.

Under laparoscopic visualization, the proximal ventricular portion of the shunt catheter was gently drawn caudally through its existing tract until the entire catheter was positioned within the abdominal cavity. Controlled traction was then applied to the segment protruding through the anus, allowing the complete catheter to pass through the preexisting sigmoid colon perforation and be extracted transanally (Video 1). The previous ventriculoperitoneal shunt system was thereby removed in its entirety. The removed system was not submitted for microbiological culture.

**Video 1:** Laparoscopic management of sigmoid colon perforation caused by a ventriculoperitoneal shunt Edited intraoperative video showing laparoscopic identification of the sigmoid colon perforation, mobilization of the previous shunt catheter into the abdominal cavity, controlled transanal extraction through the existing perforation under direct visualization, and primary closure of the colonic defect with polydioxanone sutures

The sigmoid colon defect was closed primarily by laparoscopy using polydioxanone sutures, followed by peritoneal lavage. No intra-abdominal drain was placed. Given the absence of gross peritoneal contamination, abscess formation, macroscopic peritonitis, or neurological manifestations, together with a negative cerebrospinal fluid Gram stain, a completely new ventriculoperitoneal shunt system was placed through a separate tract during the same operative session.

Empiric intravenous vancomycin, ceftriaxone, and metronidazole were initiated after blood and cerebrospinal fluid samples had been obtained. Vancomycin was discontinued after the cerebrospinal fluid culture remained negative, whereas ceftriaxone and metronidazole were continued to complete a five-day postoperative course.

Enteral feeding was resumed on postoperative day 3 and was well tolerated. The patient was discharged in good clinical condition on postoperative day 5. Follow-up evaluations at two weeks and three months demonstrated a favorable clinical course, without abdominal complications, neurological manifestations, or clinical evidence of shunt-related infection.

## Discussion

More than 100 cases of ventriculoperitoneal shunt-related bowel perforation have been described worldwide, almost exclusively through case reports and small series. Precise estimates vary because published reviews use different definitions and contain overlapping cases. Hai et al. identified 94 patients reported in the literature up to 2011, whereas Harischandra et al. subsequently identified 139 cases involving bowel migration within a broader review of distal shunt migration [1,3]. A more recent systematic review by Adel et al., using narrower criteria for bowel perforation and anal migration, included 57 cases [2]. These figures should not be added because of overlap between the study populations, but they demonstrate the exceptional rarity of this complication.

The interval between shunt placement and clinical detection is highly variable. In the systematic review by Adel et al., it ranged from 12 days to 19 years, although 55% of cases occurred within the first 12 months [2]. An earlier review reported a mean interval of 4.86 months among infants younger than one year and an overall mean of 24.8 months across all age groups [1]. Therefore, although detection in our patient occurred nine weeks after shunt placement following an initially asymptomatic postoperative period, this interval is relatively early within the reported spectrum.

Clinical manifestations range from abdominal or neurological symptoms to an entirely asymptomatic course until catheter extrusion occurs. In the bowel-migration cases analyzed by Harischandra et al., 74% of patients had abdominal manifestations and 21.5% had central nervous system manifestations [3]. Abdominal pain or tenderness, vomiting, fever, signs of peritoneal irritation, shunt dysfunction, meningitis, and ventriculitis have been reported. In the systematic review focused specifically on bowel and anal migration, transanal extrusion occurred in 82% of cases [2]. This proportion should be interpreted according to the review’s selected population and should not be extrapolated to all bowel perforations. Our patient presented with visible transanal extrusion and signs of peritoneal irritation but no neurological manifestations. Despite these findings, laparoscopy showed no feculent contamination, abscess, or macroscopic peritonitis. The limited contamination may be explained by gradual erosion and formation of a fibrous tract around the catheter, which can partially seal the bowel defect [4].

When the catheter is visibly protruding through the anus, the diagnosis is clinically evident. In less apparent cases, an abdominal radiograph or shunt series may demonstrate an abnormal catheter course, whereas contrast-enhanced computed tomography can define the perforation site and identify free air, inflammatory changes, abscesses, or collections. Endoscopy may be useful for localization and extraction in selected stable patients [4,5]. Cerebrospinal fluid Gram staining and culture are essential because the shunt may provide a route for retrograde infection by enteric microorganisms. In our patient, no preoperative imaging was obtained because the transanal catheter was directly visible and the signs of peritoneal irritation prompted urgent surgical exploration. Blood and cerebrospinal fluid samples were obtained before antimicrobial therapy.

Management must be individualized according to the patient’s stability, the presence of intra-abdominal contamination or central nervous system infection, and the condition of the affected bowel. In the systematic review by Adel et al., extraction techniques were reported for 43 patients. Direct or endoscopic transanal removal was performed in 30 cases (70%), whereas laparotomy or laparoscopy was used in six cases (14%). In another five cases (11%), an abdominal surgical approach was combined with transanal extraction [2]. Because laparotomy and laparoscopy were grouped together, the exact number of patients managed laparoscopically cannot be determined. Furthermore, no comparative studies have directly evaluated laparoscopic versus open management for this complication. The evidence supporting laparoscopy is therefore limited to individual case reports describing successful catheter extraction, primary repair or intestinal resection, and peritoneal lavage [6-8].

Despite the limited evidence, laparoscopy offers several potential advantages in selected, hemodynamically stable patients. It permits direct identification of the perforation site, assessment of the entire peritoneal cavity, exclusion of additional injuries, controlled catheter extraction, primary repair, and lavage through a minimally invasive approach. Open surgery remains appropriate when there is hemodynamic instability, diffuse or feculent peritonitis, extensive abscess formation, multiple perforations, ischemic bowel, or a need for resection or diversion. In our patient, laparoscopy identified a single 10-mm sigmoid perforation and excluded additional lesions or significant contamination. It also permitted controlled transanal extraction under direct visualization, primary suture repair, and peritoneal lavage without conversion to laparotomy or placement of an intra-abdominal drain.

A second controversial aspect is whether cerebrospinal fluid diversion should be restored immediately or in a subsequent procedure. Most published reports favor complete removal or externalization of the shunt, temporary external ventricular drainage, antimicrobial treatment, and delayed insertion of a new system after cerebrospinal fluid cultures have remained sterile [4,5]. This staged strategy reduces the risk of implanting new hardware in the presence of occult contamination and is particularly appropriate in patients with positive cerebrospinal fluid cultures, meningitis, ventriculitis, abscess formation, gross peritoneal contamination, or generalized peritonitis. Guidelines for confirmed cerebrospinal fluid shunt infection similarly recommend removal of the infected system, temporary external drainage, and delayed reimplantation according to the microorganism and serial culture results [9]. However, these recommendations were developed for confirmed or suspected shunt infection and do not specifically define the optimal approach for bowel perforation with negative initial cerebrospinal fluid studies.

Immediate replacement avoids temporary external ventricular drainage, a second anesthetic procedure, and delayed control of hydrocephalus, but it carries the risk of implanting new hardware before culture results are finalized. In our patient, the complete previous system was removed, and a new ventriculoperitoneal shunt was placed through a separate tract during the same operation. This decision was based on the absence of gross contamination, abscess formation, macroscopic peritonitis, neurological manifestations, or initial microbiological evidence of cerebrospinal fluid infection. Cerebrospinal fluid Gram staining obtained before antibiotics was negative, and cerebrospinal fluid and blood cultures subsequently showed no microbial growth. Nevertheless, a negative Gram stain alone cannot exclude infection, and final culture results were unavailable when the new system was implanted [9]. In addition, the removed shunt was not submitted for microbiological culture. Therefore, immediate replacement should be presented as an individualized strategy in a carefully selected patient rather than as a generally recommended approach.

This case adds to the limited pediatric literature by demonstrating that laparoscopy can identify the site and extent of colonic injury, facilitate controlled transanal catheter extraction, and permit primary repair of the perforation. It also illustrates the feasibility of immediate shunt replacement in a selected patient without gross intra-abdominal or microbiological evidence of infection. However, the single-patient design, the absence of culture of the removed shunt, and the three-month follow-up period prevent conclusions regarding the comparative safety of immediate versus staged reconstruction.

## Conclusions

Ventriculoperitoneal shunt-related colonic perforation with transanal extrusion can become clinically evident after an initially uneventful postoperative course. In selected hemodynamically stable patients, laparoscopy allows identification of the perforation site, assessment of intra-abdominal contamination, controlled transanal catheter extraction, and primary bowel repair. Immediate placement of a new shunt through a separate tract may be feasible when gross contamination and cerebrospinal fluid infection are absent; however, this approach must be individualized because the available evidence is limited and staged reconstruction remains preferable when infection is suspected.
